# Development of FGF-2-loaded electrospun waterborne polyurethane fibrous membranes for bone regeneration

**DOI:** 10.1093/rb/rbaa046

**Published:** 2020-10-04

**Authors:** Chi Zhang, Jianxiong Wang, Yujie Xie, Li Wang, Lishi Yang, Jihua Yu, Akira Miyamoto, Fuhua Sun

**Affiliations:** 1 Department of Rehabilitation, The Affiliated Hospital of Southwest Medical University, Luzhou 646000, P.R. China; 2 Department of Oncology, The Affiliated Hospital of Southwest Medical University, Luzhou 646000, P.R. China; 3 Faculty of Rehabilitation, Department of Physical Therapy, Kobe International University, Kobe, Japan

**Keywords:** waterborne polyurethane, fibroblast growth factor-2, emulsion electrospinning, vascularization, GBR membrane

## Abstract

Guided bone regeneration (GBR) membrane has been used to improve functional outcomes for periodontal regeneration. However, few studies have focused on the biomimetic membrane mimicking the vascularization of the periodontal membrane. This study aimed to fabricate waterborne polyurethane (WPU) fibrous membranes loaded fibroblast growth factor-2 (FGF-2) via emulsion electrospinning, which can promote regeneration of periodontal tissue via the vascularization of the biomimetic GBR membrane. A biodegradable WPU was synthesized by using lysine and dimethylpropionic acid as chain extenders according to the rule of green chemical synthesis technology. The WPU fibers with FGF-2 was fabricated via emulsion electrospinning. The results confirmed that controlled properties of the fibrous membrane had been achieved with controlled degradation, suitable mechanical properties and sustained release of the factor. The immunohistochemical expression of angiogenic-related factors was positive, meaning that FGF-2 loaded in fibers can significantly promote cell vascularization. The fiber scaffold loaded FGF-2 has the potential to be used as a functional GBR membrane to promote the formation of extraosseous blood vessels during periodontal repairing.

## Introduction

Periodontal membrane plays a decisive role in the repair and regeneration of periodontal ligaments, alveolar bone and cementum. The tissue can not only act as an effective barrier between the periodontal tissues and epithelium but also provide sufficient nutrients via a rich vascular network [1]. However, the destruction of the membrane due to injuring and disease leads to the challenge of periodontal repairing. Guided bone regeneration (GBR) membrane as a promising method has been applied to periodontal regeneration [2]. 

Some GBR membranes have been developed using polymeric materials and active biological factors. Some engineered biomimetic membranes were seeded with cells [3−5]. However, the inevitable shortcoming of the engineered GBR membrane is that seed cells often lose their ability to differentiate during *in vitro* expansion, as progenitor cells lose their properties [6]. Therefore, it is an important method to add appropriate growth factors to the environment in which cells are growing [7]. Currently, continuous controlled release of cytokines has been used in bone tissue engineering and can promote bone repair effectively [8−11]. Among them, fibroblast growth factor-2 (FGF-2) plays a crucial role in the healing process of damaged tissues. FGF-2 exists in the subendothelial extracellular matrix of the basement membrane and blood vessels, which can stimulate the activation of macrophages and promote the proliferation and differentiation of fibroblasts and endothelial cells [12], as well as the recovery of extracellular matrix and the formation of new blood vessels [13]. FGF-2 can accelerate wound closure by stimulating the proliferation of mesenchymal stem cells [14−16]. In addition, FGF-2 can stimulate the synthesis of collagen and the division of fibroblasts, vascular endothelial cells and keratinocytes, which is conducive to the formation of granulation tissue and epidermal regeneration *in vivo* [17, 18]. FGF-2 is highly expressed in the early stages of fracture healing [19]. However, keeping the activity of biological factors is still a big challenge.

Electrospinning is a promising technology to fabricate fibrous membrane with high surface area to volume ratio and high porosity. Therefore, many polymers have been electrospun into fibers serving as drug carrier or shielding membrane for isolation of peripheral soft tissue and avoiding scar tissue formation [20]. Nevertheless, various organic solvents are usually used, such as trifluoroethanol (TFEA) [21], 1,1,1,3,3,3-Hexafluoro-2-propanol (HFIP) [22], tetrahydrofuran (THF) [23], etc., which are difficult to completely remove. But the residual solvent usually has many negative effects on biological factor. To keep the activity of factor, the water is an ideal electrospinning medium. However, there are only a few water soluble polymers suitable for electrospinning. Emulsion electrospinning technology has been developed to fabricate polymer fibers using water [24, 25]. Waterborne polyurethane (WPU), with water as the dispersion [26], has gradually replaced the traditional solvent-based polyurethane because of low viscosity, nontoxic, pollution-free, low cost, good applicability and safety [27, 28]. However, the pure WPU emulsion is not suitable for electrospinning because of the weak cohesive interparticle forces. So, emulsion electrospinning usually needs a template polymer to provide viscoelasticity and help the formation of fiber [29]. WPU has been fabricated into fibrous membrane but the sustained release of factor and application in periodontal repairing is rarely studied [24].

In this study, a new biodegradable WPU was synthesized by using poly(ε-caprolactone) (PCL) as soft segment, isophorone diisocyanate (IPDI) as hard segment, 2,2-dimethylpropionic acid (DMPA) and l-lysine as chain extender. The WPU was chosen as a biomaterial because of its excellent properties, e.g. nontoxic, high elasticity and malleable. Polyethylene oxide (PEO), a water soluble polymer as viscosity enhancer, is used to integrate WPU emulsion into fibers. To improve the mechanical properties, elasticity, dimensional stability and solvent-resistance of WPU, trimethylolpropane-tris-(3-aziridinyl propionate) (XR-100) was chosen as the crosslinking agent. The emulsion electrospinning method was used to fabricate the WPU into fibrous membranes loaded FGF-2 with the functionalities of promoting angiogenesis. In addition, the structural characteristics, physicochemical properties and biological activities of the fibrous membrane before and after the loading of the factor were examined, and the controlled release behavior of the biological factor and its effect on cell growth were evaluated.

## Materials and methods

### Synthesis of WPU

WPU was synthesized via a three-step method. Briefly, IPDI (Shanghai Aladin Co. Ltd, China) and Polycaprolactone diol 2000 (PCL2000, *M*_n_ = 2000, Sigma-Aldrich, USA) at the molar ratio of NCO/OH of 2/1 were pre-polymerized in a three-necked flask in a nitrogen atmosphere with mechanical stirring, and the reaction was continued at 80°C for 2 h to obtain the prepolymer. Then DMPA (Shanghai Aladin Co. Ltd) was added as a chain extender to the prepolymer under thorough stirring for 2 h after cooled to 60°C, a certain amount of diethylamine was added to neutralize the carboxyl group in the reaction system. Finally, the chain-extending product was slowly dropped into the lysine (l-lysine, Shanghai Aladin Co. Ltd)/H_2_O solution, and the stable WPU product was obtained after mechanical stirring for 2 h. The solid content of WPU emulsion was 30% w/v and stored at room temperature for further use. The schematic diagram of the polymerized procedure was shown in Supplementary Fig. S1.

### Preparation of FGF-2 loaded WPU fibrous membranes

PEO (*M*_w_ = 100 000, Sigma-Aldrich) was dissolved in certain degree diluting WPU emulsion to obtain a 30% w/v electrospinning solution. The concentration of PEO was 6% w/v (WO6). Then the crosslinking agent trimethylolpropane-tris-(3-aziridinyl propionate) (XR-100, Hubei Xinmingtai Chemical Co. Ltd, China) was added in WPU solution with the mass ratio to WPU were 3 wt% (C3) and 5 wt% (C5), respectively. FGF-2 was added to the electrospinning solution with the mass ratio to polymer 0.2 μg g^−1^ (WO6-0.2, C3-0.2, C5-0.2) and 2 μg g^−1^ (WO6-2, C3-2, C5-2), respectively.

**Figure 1.**
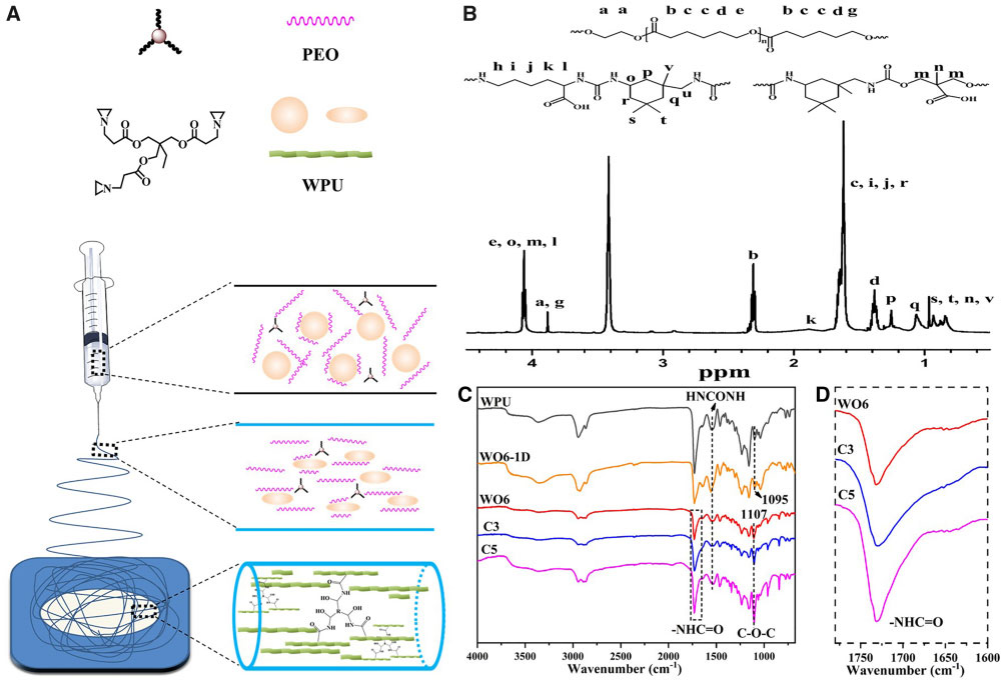
(**A**) The electrospinning process of WPU emulsion, and the mechanism for the formation of WPU fibers via emulsion electrospinning. (**B**) ^1^H NMR spectra of synthetic WPU. (**C**) At-FTIR spectra of WPU casting film, WO6, C3 and C5 fibrous membranes. (**D**) The magnified image of spectra of NHCOO.

The FGF-2 (PeproTech Co. Ltd, USA) loaded membranes were fabricated via ‘emulsion electrospinning’ method. The electrospinning liquids were placed into the syringe with 22# blunt needles (the diameter is 0.4 mm). A constant volume flow rate of 0.5 ml h^−1^ was maintained. A high voltage of 15 kV was applied when the solution was drawn into fibers. And fibers were collected by an aluminum foil covered on the collection plate at a distance of 15 cm from the needle tip. The electrospinning process and the mechanism for the formation of WPU fibers were shown in Fig. 1A.

In order to observe the fiber morphology after removing the excipient PEO, WO6, C3 and C5 fibrous membranes were immersed in deionized water for 24 h and washed three times. Then the samples were obtained after freeze-dried named RWO6, RC3 and RC5, respectively. The process of synthesis of WPU fibers is shown in Table 1.

**Table 1.** The electrospinning solution of each group via emulsion electrospinning

	WPU casting film	Electrospun fibrous membranes							
		WO0^a^	WO3^a^	WO6^a^	C3^b^	C5^b^	RWO6	RC3	RC5
WPU (w/v%)	30	30	27	24	24	24	24	24	24
PEO (w/v%)	0	0	3	6	6	6	6	6	6
XR-100 (wt%)^c^	0	0	0	0	3	5	0	3	5
Removing PEO	−	−	−	−	−	−	√	√	√

a The numbers in the abbreviation of WO0, WO3 and WO6 mean the mass fraction of PEO.

b The numbers in the abbreviation of C3 and C5 mean the mass ratio of XR-100 to WPU.

c The concentration of XR-100 is the mass ratio of XR-100 to WPU.

The drug effectively embedded in the WPU fibrous membranes was determined by the ELISA kit. Briefly, the drug-loaded WO6-0.2 and WO6-2 fibrous membranes (5 mm×5 mm, *n* = 5) were cut into fibrous fragments. Then these fibers were scattered in PBS solution (pH = 7.4) with 1 wt% of bovine serum albumin (Hyclone, USA) and incubated for 24 h in a 37°C constant temperature shaker. The culture PBS was collected and replaced every 8 h using centrifugation. Finally, the FGF-2 content was measured according to the instruction of ELASA kit. The drug-loading efficiency reached ∼95% in different fibrous membranes determined by Equation (1). The resulting drug-loaded fibrous membranes were sterilized by ethylene oxide at low temperature and stored at −20°C.

(1) $$\mathrm{Drug}-\mathrm{loading}\;\mathrm{efficiency}\;(\%)=\frac{m_{\text{loaded}\;\text{drug}}}{m_{\text{total}\;\text{drug}}}\times 100$$

### Characterization

#### Nuclear magnetic resonance spectroscopy

WPU casting film was prepared at room temperature and dried in an oven at 70°C sufficiently. The WPU film was dissolved in deuterated chloroform and its ^1^H spectrum was analyzed by 600 MHz nuclear magnetic resonance spectrometer (NMR, Bruker, Switzerland).

#### Scanning electron microscope

The fibers were collected on aluminum foil. The membranes were cut into 5 mm × 5 mm and gold sputter-coated. Then the samples were observed by the scanning electron microscopy (SEM, JSM-6510LV, JEOL, Japan). Image Pro Plus software was used to analyze the diameters of a total of 100 fibers in different SEM images, and the diameter distributions of each group of samples were obtained through statistical analysis.

#### Fourier transform infrared spectroscopy

The infrared spectral analysis of fibrous samples was performed in an attenuated total reflectance-Fourier transform infrared spectrometer (ATR-FTIR, Nicolet 6700, Thermo Fisher Scientific, USA). The spectra were collected at a spectral resolution of 4 cm^−1^ and a scanning range of 675 − 4000 cm^−1^.

#### Mechanical testing

The mechanical properties of the samples were tested using a Universal Testing Machine (AG-IC 50KN, SHIMADZU, Japan). The dumbbell specimens were cut from the membranes into dimensions of 4 × 50 mm. Conditions, the testing was carried out at a speed of 25 mm min^−1^ at room temperature with a load cell capacity of 250 N. At least five specimens were tested and the reported data were the average of five independent samples.

#### *In vitro* degradation

The WPU film, C3 and C5 fibrous membranes (10 mm × 10 mm, *n* = 5) were put into 5 ml PBS solution and then incubated with shaking for a certain time (1, 2, 3, 7, 14 and 21 days) at 37°C. After incubation, the samples were taken out and washed with distilled water and dried in a vacuum oven at 37°C to a constant weight. The morphology of each group of degradation samples was observed by SEM.

#### FGF-2 release study

The pre-weighted drug-loaded fibrous membranes (WO6-0.2, WO6-2, C3-0.2, C3-2, C5-0.2 and C5-2) were cut into 5 mm × 5 mm specimens (*n* = 3) and placed in a PBS solution (pH = 7.4) containing 1 wt% of bovine serum albumin. Then these membranes were incubated at 37°C in a constant temperature shaker for a certain time (1, 2, 3, 7 and 21 days). Accurately remove 1 ml of the release solution at the scheduled time point while replenishing the same amount of fresh incubation solution. The collected liquid was stored at −20°C, and the content of FGF-2 released at each stage was measured via ELISA kit.

#### *In vitro* study

Rat mesenchymal stem cells (rMSCs) were isolated according to the previous study [30]. Primary rMSCs were aspirated from femurs and tibiae removed from SD rats (3–4 weeks old, ∼100 g) purchased from the West China Center of Medical Sciences of Sichuan University (Sichuan province, China). The harvested bone marrow suspension was mixed with α-MEM medium (Gibco, 1 ml per well) with 10% fetal bovine serum (FBS; Hyclone) and cultured in a humidified incubator (37°C, 5% CO_2_). The culture medium was replaced every 3 days. rMSCs at the third passage were utilized for the experiments.

The fibrous membranes were cut into square samples (10 mm × 10 mm) sterilized with ethylene oxide at a low temperature according to GB18279-2000. The samples were cocultured with rMSCs (2 × 10^4^ cells well^−1^) in 24-well plates compared with the blank control (tissue culture plastic, TCP), which was equilibrated in α-MEM medium (Gibco, 1 ml well^−1^) with 10% FBS in a humidified incubator (37°C, 5% CO_2_).

The proliferation activity of cell-seeded on the samples was detected by CCK-8 assay after 1, 4 and 7 days. The optical density at 450 nm was obtained via a microplate reader (PerkinElmer, USA). The cell viability was determined by fluorescence staining, as performed on, respectively. The cells seeded on the fibrous membrane for 4 and 7 days were rinsed with PBS and labeled with the live/dead reagent (LIVE/DEAD Viability/Cytotoxicity Kit, Life Technologies, USA).

The morphology and the spreading of the rMSCs seeded on the membranes were observed with fluorescence microscopy (TE 2000-U, Nikon Eclipse, Japan). Before the fluorescence observation, the samples with cells after cultured 7 days were labeled with Mito Tracker^®^ Green FM, Alexa Flour^®^ 546 phalloidin, and Hoechst33342 (Life technologies).

The blood covers (Sailing brand, nontoxic glass) sterilized by high temperatures were placed in a six-well plate. Then rMSCs were inoculated on the blood covers at the density of 1 × 10^4^ cells ml^−1^. The drug-loaded membranes (C3-0.2 and C3-2, 15 mm × 15 mm) were placed in the up-well. rMSCs cocultured with α-MEM containing 5 ng ml^−1^ FGF-2 was used as the positive control (PC) group. The cell-seeded blood covers were rinsed with PBS after 14 days, and cell slides were made. For Immunohistochemical staining of CD31 and VEGF, ECs slides of angioblast-induced cells were labeled with anti-CD31 monoclonal antibody and mouse anti-rat VEGF, respectively. The samples were obtained after routine dehydration, transparency and tablet sealing.

### Statistical analysis

The experimental data were processed using SPASS v17.0 and all data were expressed as mean ± variance (SD). Student’s *t*-test was used to compare two groups. *P *<* *0.05 indicated that the result was statistically significant, and *P* < 0.001 was extremely significant values.

## Results

### Chemical properties of the WPU

As shown in Fig. 1B, the peaks observed from 0.8 to 1.0 ppm represented the −CH_3_ group in IPDI and DMPA. The signals at 1.25 and 1.06 ppm were attributed to the methylene group in the IPDI. The peak at 1.22 ppm related to the methylene groups in hard-segment lysine and soft-segment PCL. The peaks at 3.88, 2.30 and 1.38 ppm were attributed to PCL.

The infrared spectra of the WPU casting film, WO6, C3 and C5 fibrous membranes were displayed in Fig. 1C. The peak at 2270 cm^−1^ belonged to the NCO group was not observed, indicating that the NCO group had been completely reacted. The strong peaks appearing at 3300 and 3400 cm^−1^ were assigned to −NH in WPU. The signals at 1730 and 1531 cm^−1^ were attributed to the NHC=O group and NHCONH in polyurethane. The peaks at 1095 and 1107 cm^−1^ belonged to the −C−O−C− groups in WPU and PEO molecules, respectively. Stronger peak intensity, which was representative of the increased content of ether content in the fibrous membrane. The peak intensity of the NHC=O group increased gradually with the increase of the crosslinking agent in Fig. 1D, which should be driven by the formation of the new −NHC=O group reacted between the crosslinker and the carboxyl group of polyurethane.

### Morphology of fibers

**Figure 2.**
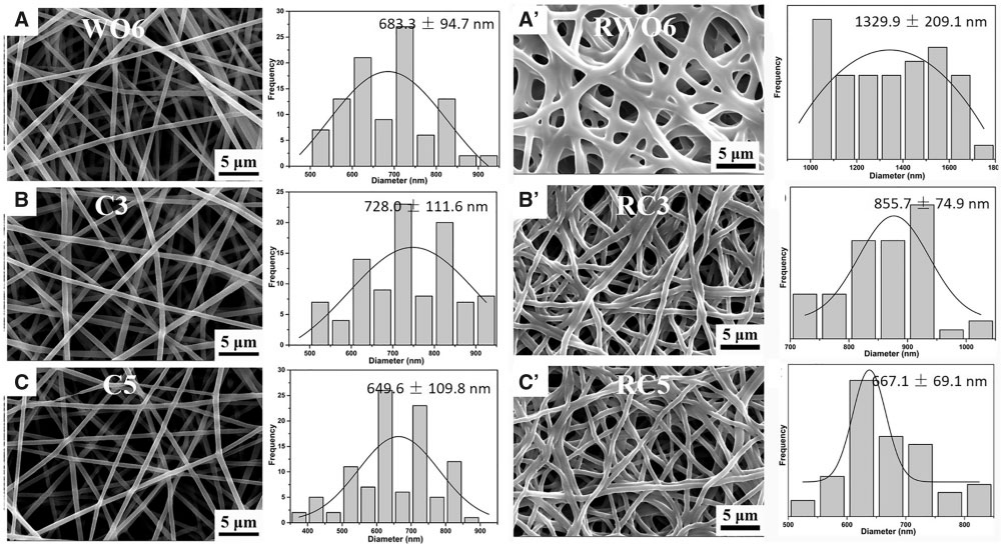
SEM images, diameter distribution and average diameters (*n* = 100) of the WPU fibers before and after washing treatment.

To investigate the effect of PEO on the formation of WPU fibers, the samples with different PEO concentration were collected and detected with SEM, as shown in Supplementary Fig. S1. The microstructure, diameter distribution and average diameter of WO6, C3 and C5 fibrous membranes were shown in Fig. 2A−C. The content of XR-100 had no obvious influence on the morphology and diameter distribution of fiber.

To investigate the effect of crosslinker on waterproof, the morphology of WPU fibers after removed the PEO with deionized water was shown in Fig. 2A′−C′. Flabby fibers were visible after washed, and the surface of fibers kept smooth. The WO6 fibers swelled obviously and the average diameter of RWO6 fibers was about twice that of WO6 fiber (*P *<* *0.001). RWO6 fibers fused and formed physical crosslinking point. The diameter of RC3 fiber increased significantly compared with C3 fiber, and the diameter increased from 600 − 850 to 800 − 950 nm (*P *<* *0.001). The adjacent RC3 fibers bonded together, but did not fuse. C5 fibers adhered to each other slightly, and there was no significant difference in average diameter between C5 and RC5 fibers, indicating that crosslinked C5 fibers do not swell significantly after PEO excipient was removed.

### *In vitro* degradation

**Figure 3.**
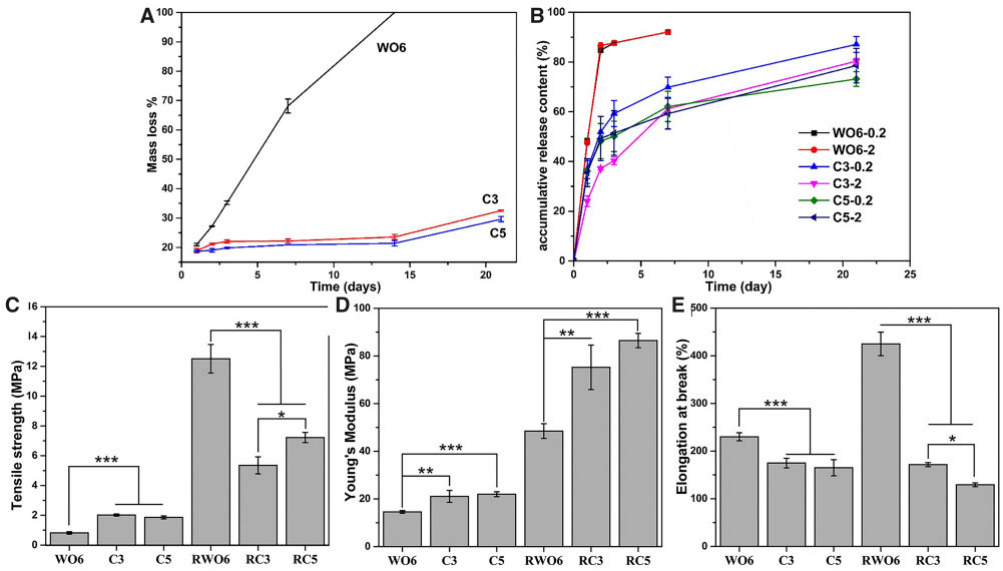
(**A**) The degradation performance of WO6, C3 and C5 fibrous membranes (*n* = 3). (**B**) The kinetic plot of FGF-2 release from different drug-loaded fibrous membranes (*n* = 3). (**C**) Tensile strength, (**D**) Young’s modulus and (**E**) elongation at break of WO6, C3, C5, RWO6, RC3 and RC5 fibrous membranes (*n* = 5, ***P < 0.001, **P < 0.01, *P < 0.05).

Figure 3A illustrated the mass loss of WO6, C3 and C5 fibrous membranes during incubation in PBS solution. As can be seen, the degradation of the WO6 fibers was the fastest and they are degraded completely within 2 weeks. The mass loss of WO6 fibers was ∼20 wt%, mainly due to the dissolution of PEO after rinsed for 1 day. Then WO6 fibrous membrane broke into fibrous fragments after 1 week (Supplementary Fig. S3). After the crosslinking treatment, the degradation rate of C3 and C5 membranes was slower than that of WO6 fibers, and the mass loss was up to 20 wt% after 3 days due to the dissolution of PEO. The biodegradable speed of C3 fibers was similar to the C5 fibers when incubated *in vitro*. The results indicated that crosslinker slowed down the degradation rate of WPU fibers obviously.

### Drug release behavior

The *in vitro* release of FGF-2 was determined using an ELISA kit. As shown in Fig. 3B, the release rate of FGF-2 in WO6 fibrous membrane was burst on the 1st day of the 21-day release test. After 7 days, the WO6 fibers were severely broken (Supplementary Fig. S3), and FGF-2 had released ∼92% of the total loading. The release of FGF-2 loaded in crosslinked fibers decreased significantly. The cumulative release amount was 61 − 70% on the 7th day and the cumulative release amount on the 21th day was 73 − 87%. The release trend of high and low drug-loaded fibers was basically consistent. There was a significant difference between the two groups of drug-loaded fibers crosslinked with 3% XR-100, while there was a difference between the two groups of drug-loaded fibers with a high crosslinking agent at only 21 days (*P* < 0.05).

### Mechanical properties

Comparing to WO6, C3 and C5 membranes, the tensile strength and Young’s modulus of WO6 fibrous membrane were the least, and its strain at break was the largest, the tensile strength values of C3 and C5 fibrous membranes were ∼2.5 times than that of WO6 fibrous membrane (*P *<* *0.001) (Fig. 3C), while there is no significant difference between C3 and C5. The strength of the fibers increased significantly after PEO was removed from the fibers. The tensile strength of the RWO6 fiber membrane is about two times than that of RC3 and 1.5 times that of RC5. The modulus values of C3 and C5 are ∼1.5 times than that of WO6, but RC3 and RC5 fiber membranes, and a similar trend are observed in Young’s modulus of fibers removed PEO (Fig. 3D). The elongation at break of WO6 fibers is 1.3 times than that of C3 and C5 (*P *<* *0.001). After removing the PEO, the elongation at break of the fibers increased obviously. RWO6 was 2.5 times than that of RC3 and three times than that of RC5, respectively (*P *<* *0.001) (Fig. 3E). The results indicate that the mechanical properties of WPU fibers could be controlled by regulating the amount of crosslinker.

### Cell proliferation and viability

**Figure 4.**
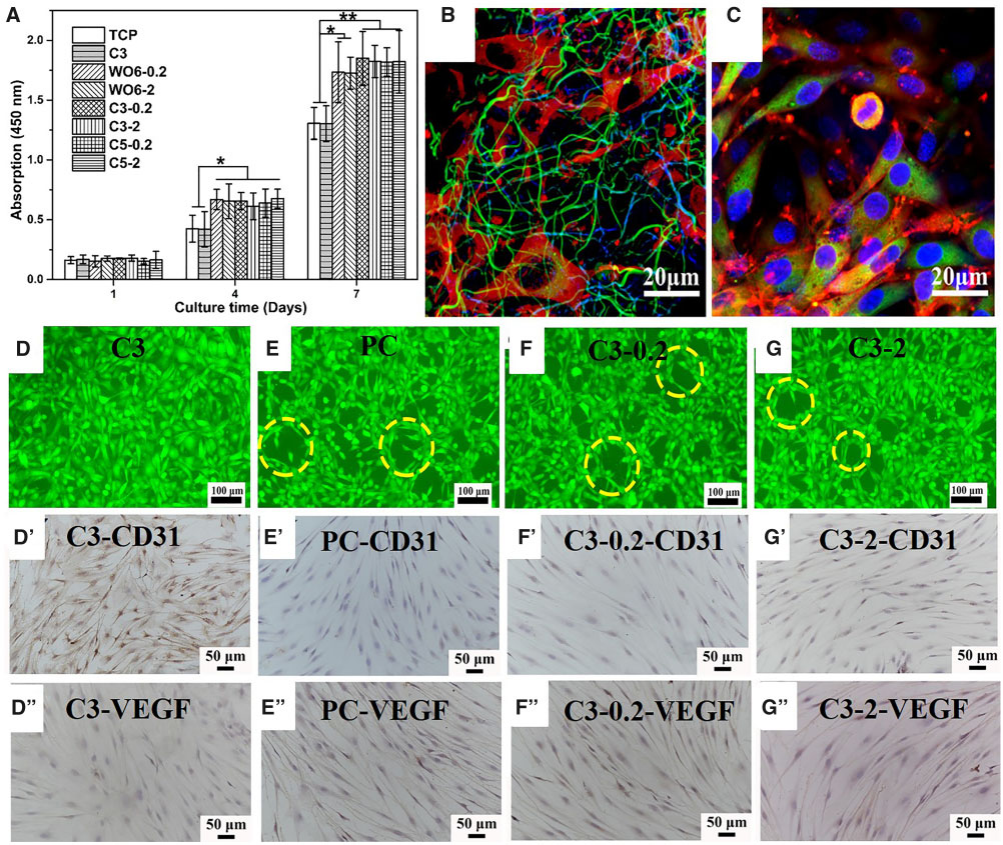
(**A**) Cell proliferation of rMSCs cultured on fibers loaded with FGF-2 (*n* = 5, **P < 0.01, *P < 0.05). Confocal images of rMSCs after 4 days of culture in (**B**) C3 fibers and (**C**) WPU casting film. Fluorescent images (live/dead staining) of co-cultured cells on the well for 7 days: (**D**) C3, (**E**) positive control group (PC), (**F**) C3-0.2 and (**G**) C3-2. Light microscopy photographs of immunohistochemical staining cells co-cultured with (**D**′, **D**″) C3, (**E**′, **E**″) PC, (**F**′, **F**″) C3-0.2, (**G**′, **G**″) C3-2 fibrous membranes for 7 days. The long spindle cells in yellow circles connected end to end into a ‘capillary’ pattern.

As shown in Supplementary Fig. S4, no obvious influence of XR-100 on the proliferation of cells on WPU fibers. The proliferation of rMSCs on the drug-loaded fibers was shown in Fig. 4A. No significant difference between the control group and fibrous membranes on the first day, indicating that the FGF-2 has no significant effect on the adhesion of rMSCs. After co-cultured for 4 days, the number of cells on drug-loaded fibers was significantly higher than that on the control group (TCP and C3) (*P *<* *0.05), indicating that released FGF-2 promoted cell proliferation significantly. However, there was no significant difference between different fibrous membranes loaded 0.2 and 2 μg g^−1^ FGF-2. The results indicated that the load concentration of 0.2 g g^−1^ had reached the threshold content of FGF-2. After 7 days of culture, there were more cells on the crosslinked fiber experimental group (C3-0.2/2 and C5-0.2/2) than that on the control group (*P *<* *0.01). However, the number of cells on WO6 group due to the burst release of FGF-2 in the early stage.

### Cell morphologies

To more visually observe the spreading behavior of the rMSCs on different membranes, the cells were stained for 4 day with Hoechst, for the nuclei of the cells, Green FM for mitochondria and TRITC-phalloidin for the cytoskeletal actin, to reflect the difference of cell attachment and cytoskeleton shape on cast WPU film and C3 fibrous membrane. As shown in Fig. 4B and C, the rMSCs on C3 fibers membrane with a large number of microfilaments connecting to each other and infiltrating into the fiber. However, the rMSCs on WPU film presented more spindle shape and were adjacent to each other, which seriously inhibited spreading. The results indicated that this 3D fibrous structure was obviously favorable for the spread of cells than smooth surface of WPU casting film. Also, the SEM images with a larger magnification provided a more clear characterization of cellular spreading after cultured 4 days, as shown in Supplementary Fig. S5.

### Cell differentiation

To evaluate the effect of FGF-2 released from the drug-loaded fibrous membranes on the differentiation behavior of rMSCs, the morphology, CD31 and VEGF expression measurements are essential. As shown in Fig. 4D, the C3 fibers had no significant effect on the morphology of rMSCs with the polygonal shape on the well plates. The morphology of the C3-0.2 and C3-2 groups was similar to that of the PC group, and the long spindle cells connected end to end into a ‘capillary’ pattern, as shown by the circle in Fig. 4E−G.

The expression of CD31 and VEGF, as the characteristic proteins of vascular endothelial cells, was determined via immunohistochemical staining, as shown in Fig. 4D′−F′ and D″−F″. The cells on C3-0.2 and C3-2 fibrous membranes presented elongated filaments. The CD31 and VEGF of C3-0.2 and C3-2 expressed positively like the cell phenotype of the PC group, while cells in the negative control group (C3 fibrous membrane) did not express the two factors. The results indicated that the release level of FGF-2 from the fibers could meet the required concentration for rMSCs to differentiate into vascular endothelial cells.

## Discussion

The angiogenic function of periodontal membrane plays a vital role in alveolar bone regeneration. Developing tissue-engineered membrane is a comparatively promising method. Electrospinning, as a promising method, has been used to fabricate fibrous scaffolds similar to natural ECM structure [31, 32]. Synthesis materials have been used as a tissue-engineering graft due to their mechanical properties and biodegradation [21, 33]. WPU has been used in the field of bone tissue engineering, because of its controlled degradation by crosslinking, nontoxic and microdissection phase properties similar to natural collagen fibers [34]. Inspired by the vascularization and structure of periodontal membrane, we incorporate biological factors into the fibers to facilitate the proliferation and angiogenic differentiation of cells.

A new degradable WPU was designed and prepared as the first step in this study. Considering the rapid degradation of ‘emulsion electrospinning’ WPU fibers, a chemical crosslinking agent was further used to form a chemical bridging point between the molecular chains. The crosslinking structure was used to increase the interaction between the polyurethane molecules, and the accumulation between the molecular chains became closer. This crosslinking structure and the phase separation characteristics of polyurethane molecules lead to better water-resistance [35]. *In vitro* degradation, the PEO was dissolved into the PBS from the fiber firstly, and then the entanglement between the WPU molecules become loosen. However, the network structure of intermolecular chemical crosslinking inhibits the immersion of H_2_O molecules into the crosslinked fibers. At the same time, the degradation rate obviously slowed down due to the crosslinked structure. Crosslinking modification is conducive to the formation of interpenetrating network structure among polyurethane molecules (Fig. 1), which hinders the slip between molecules and weakens the ductility of polyurethane fibers. At the same time, the crosslinking agent and the carboxyl group in the polyurethane molecule form more high-polarity groups, which increases the mechanical strength, Young’s modulus, and rigidity of the molecular chain (Fig. 3C−E). Comparing the fiber morphology before and after removing PEO (Fig. 2), the fibers fused at the binding point in different degrees, which increase the tensile strength significantly due to the increase of crosslinking point of the fibrous membrane. In addition, the strength of RWO6 was higher than that of RC3 and RC5 because of the higher degree of fusion in RWO6 fibers after removed PEO (Fig. 3C). Crosslinking treatment also increases the content of the crosslinking points and polar groups (−NHC=O) in the molecules (Fig. 1D), which increases the hydrogen bond and makes the molecules more difficult to move, so the modulus increased significantly. The modulus increased to 90 MPa, which can match that of protein fiber (MPa−GPa) in natural tissues [36]. The mechanical properties of the drug-loaded fibrous membrane matched with the need and design of bionic periodontal tissue.

In addition to vascularization capability, as a widely used bioactive factor, FGF-2 is a potent angiogenic protein, which promotes the proliferation and differentiation of mesenchymal cells [37−39]. Moreover, it also involves the synthesis of collagen in the early stages of fracture healing [18]. However, high dose FGF-2 was usually used due to the short biological half-life, which could cause a potential side-effect. Therefore, WPU fibers as a drug delivery system for keeping activity and controlled release of FGF-2 was synthesized successfully. Through the emulsion electrospinning technology, we created the drug-loaded micro-/nano-fibrous WPU membrane with controlled mechanical, degradable and drug-released property. As shown in Fig. 2A′, the uncrosslinked WO6 electrospinning fibers swell easily in solution. The diameter of RWO6 swelling is two times than that of WO6. The loosen structure and the infiltration of large amount solution lead to the unifacial burst release of FGF-2. However, the reaction between the crosslinking agent XR-100 and the carboxyl of WPU molecules in C3 and C5 fibers results in the formation of network structure. So, the crosslinking fibrous and molecular structure change difficultly when immersed in PBS due to the restraint of the solution penetrating the fibers. The crosslinked structure delays the release rate of FGF-2 enveloped in the fibers, and achieve the long-term sustained release of the drug. Compared with other entrapment carriers, the fibrous structure can still maintain stability due to the crosslinked fibers when soaked for 21 days, and the high-crosslinked drug-loading fibers group achieved long-term release for nearly 21 days owing to the high waterproof performance (Fig. 3B).

Considering the experimental data *in vitro*, the mechanism of the fibrous scaffold as bionic periodontal membrane used in bone regeneration lay in the vascularization by controlling release FGF-2. Firstly, the mesenchymal stem cells were isolated from femurs and tibiae. The drug-loaded bionic membrane not only provided a microenvironment for the adhesion, proliferation and differentiation of cells, but also promote the vascularization of fibrous membrane applied in bone regeneration. Comparing the growth state of the cells on the fibrous membrane and the cast film, it was found that the cells spread better on the fibrous surface, as shown in Fig. 4B. The fibrous environment like ECM can be adjusted according to the needs of cell growth, and also feedback the changes in cell signals and functions [40]. In addition, the 3D environment of the fiber structure is more conducive to cell adhesion than the 2D plane of the casting film. The immunohistochemical expression of angiogenic-related factors was positive, meaning that FGF-2 loaded in fibers can significantly promote the cell vascularization. Besides, the loaded concentration of FGF-2 at 0.2 μg g^−1^ meet the excitation threshold for differentiation of rMSCs. The results showed that the drug-loaded fibrous membrane could promote the proliferation and require the differentiation of bone marrow mesenchymal stem cells into vascular endothelial cells. Overall, the drug-loaded bioresorbable bionic membrane might be a suitable candidate material for bone regeneration as GBR membrane.

The performance of the fibrous membranes was evaluated and the results confirmed that controlled properties of the fibrous membrane had been achieved with controlled degradation, suitable mechanical properties and sustained release of the factor for periodontal regeneration. Based on the high vascularity of periodontal membrane, combined with its fibrous structure, we proposed a bionic membrane that can simulate the vascularization course of natural periodontal tissue. Whether in material properties or *in vitro* performance, the ability of GBR membrane was in accordance with the hypothesis. However, as a promising bio-scaffold to solve clinical problems, more studies are needed to be done. The concentration of FGF-2, the degree of crosslinking and the microstructure such as the diameter and porosity of fibers may cause alteration in cytokine release, cell adhesion, transfer of bioactive factors, the proliferation of cells or other aspects, which need further optimization to seek the best results. Further *in vivo* experiment should be done because the effectiveness and predictability of the sustained release drug-loaded GBR membrane need be evidenced in promoting the revascularization and periodontal regeneration. Therefore, based on this research, further improvement is to be made on the current GBR membrane due to its distance from a clinically competent one.

## Conclusions

In summary, inspired by the vascularization and fibrous structure of periodontal membrane, we created a sustained release FGF-2-loaded WPU fibrous membrane as a promising artificial membrane for periodontal repair via emulsion electrospinning technology. A series of WPU fibrous membranes with different concentrations of crosslinker and FGF-2 have been successfully fabricated. The sustained release and controlled mechanical properties were characterized by the detection of the activity of FGF-2 and the tensile property, which represented the mechanisms of molecular movement and structure, respectively. Furthermore, the vascularization of bionic membrane was determined by the proliferation and angiogenic differentiate mechanism of rMSCs. Overall, in this study, the sustained release drug-loaded GBR membrane may be a promising candidate material for clinical treatment of periodontal defect with the periosteal injury.

## Supplementary data

Supplementary data are available at *REGBIO* online.

## Funding

This work was supported by the Doctoral Research Initiation Fund of Affiliated Hospital of Southwest Medical University, University Sponsored Research Program of Southwest Medical University (2019ZQN097) and the National Natural Science Foundation (81802246).

*Conflict of interest statement*. None declared. 

## Supplementary Material

rbaa046_Supplementary_Data
