# Supplementary material for: Development of FGF-2-loaded electrospun waterborne polyurethane fibrous membranes for bone regeneration
Source: Regen Biomater. 2020 Oct 4;8(1):rbaa046. doi: 10.1093/rb/rbaa046 (PMC7947599; doi:10.1093/rb/rbaa046)
Supplement: rbaa046_Supplementary_Data [file rbaa046_supplementary_data.docx]

Supporting Information

**Development of FGF-2-loaded electrospun waterborne polyurethane fibrous membranes for GBR membrane**

Chi Zhang ^a^, Jianxiong Wang ^a^, Yujie Xie ^a^, Li Wang ^a^, Lishi Yang ^b^, Jihua Yu ^a^, Akira Miyamoto ^c^, Fuhua Sun *^a^

^a^ Department of Rehabilitation, The Affiliated Hospital of Southwest Medical University,

Luzhou 646000, P. R. China.

^b^ Department of Oncology, The Affiliated Hospital of Southwest Medical University,

Luzhou 646000, P. R. China.

^c^ Department of Physical Therapy Faculty of Rehabilitation of Kobe International University, Japan.

**Characterize**

1. Particle size and Zeta potential analysis

The particle size and zeta potential of WPU emulsion, WO3, and WO6 electrospinning solutions were measured using a particle analyzer (Zeta sizer Nano ZS, Malvern Instruments, UK).

2. Electrospinning liquid viscosity

The rotational viscosity of WPU emulsion, WO3, and WO6 electrospinning liquid was measured by the DV-79 digital viscometer (Shanghai precision instrument co., Ltd.). About 10 mL of sample solution was injected into the sample cup at room temperature, and the viscosity value of each sample was obtained at a rotational speed of 75 rpm.

3. SEM morphology of cells on fibers

The morphology and spreading of the rMSCs seeded on the membranes were observed with SEM (JSM-6510LV, JEOL, Japan). For the SEM observation, the fibrous membranes co-cultured with the cells for four days were rinsed with PBS, fixed with glutaraldehyde, dehydrated with graded tertbutanol/ethanol solutions, freeze-drying, and gold sputter-coated.

**Results**


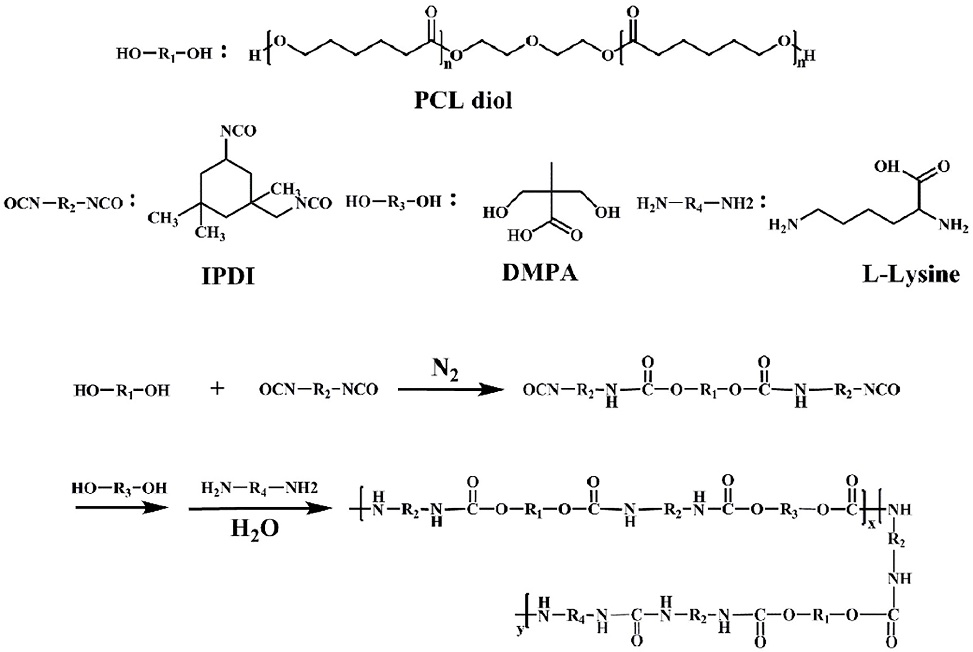


Figure S1 The chemical structure of components and schematic diagrams of the polymerized procedure of WPU

Table S1 – The particle size, Zeta potential and viscosity of electrospinning solution (*n* = 3)

| samples | Particle size/nm | Zeta potential/mV | Viscosity/mPa • S |
| --- | --- | --- | --- |
| WO0 | 72.0 ± 2.3 | -38.3 ± 0.9 | 81 ± 8.5 |
| WO3 | 87.7 ± 3.1 | -33.0 ± 1.0 | 224.7 ± 11.9 |
| WO6 | 102.6 ± 3.6 | -30.6 ± 0.6 | 1160 ± 384.3 |


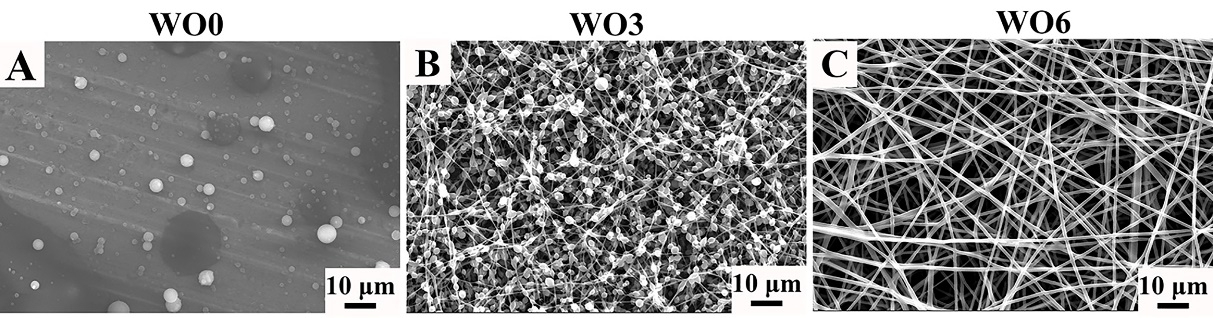


Figure S2 SEM images of electrospun WO0, WO3, and WO6 samples

As shown in Table S1, the particle size, zeta potential, and viscosity of different electrospun solutions were detected to investigate the effect of PEO on the formation of WPU fibers. The diameter of polymer particles in the emulsion and the viscosity of electrospinning solution increase obviously with the increase of PEO concentration. However, the surface potential of emulsion particles decreases due to the wraparound of PEO molecule.

Figure S2 shows the SEM morphology of electrospun samples with different amounts of PEO. The WO0 electrospinning solution without PEO only realized electrospray, and WPU microspheres were obtained (Figure S2A). The molecular chain of the WPU curled into particles and could not form a continuous phase in the aqueous solution. Under the high voltage of electrospinning, the emulsion particles agglomerated and entangled to form microspheres by electrospray. When 3 w/v% PEO was added, few of PEO hydrophilic long molecular chains dispersed in the emulsion, which can be pulled to form fibers during the electrospinning process. However, most of the particles could not be wrapped and pulled by the PEO molecular chain to form continuous fiber, and many beads and independent WPU microspheres formed. When the content of PEO was 6 w/v%, a uniform fiber structure can be obtained under the experimental conditions.


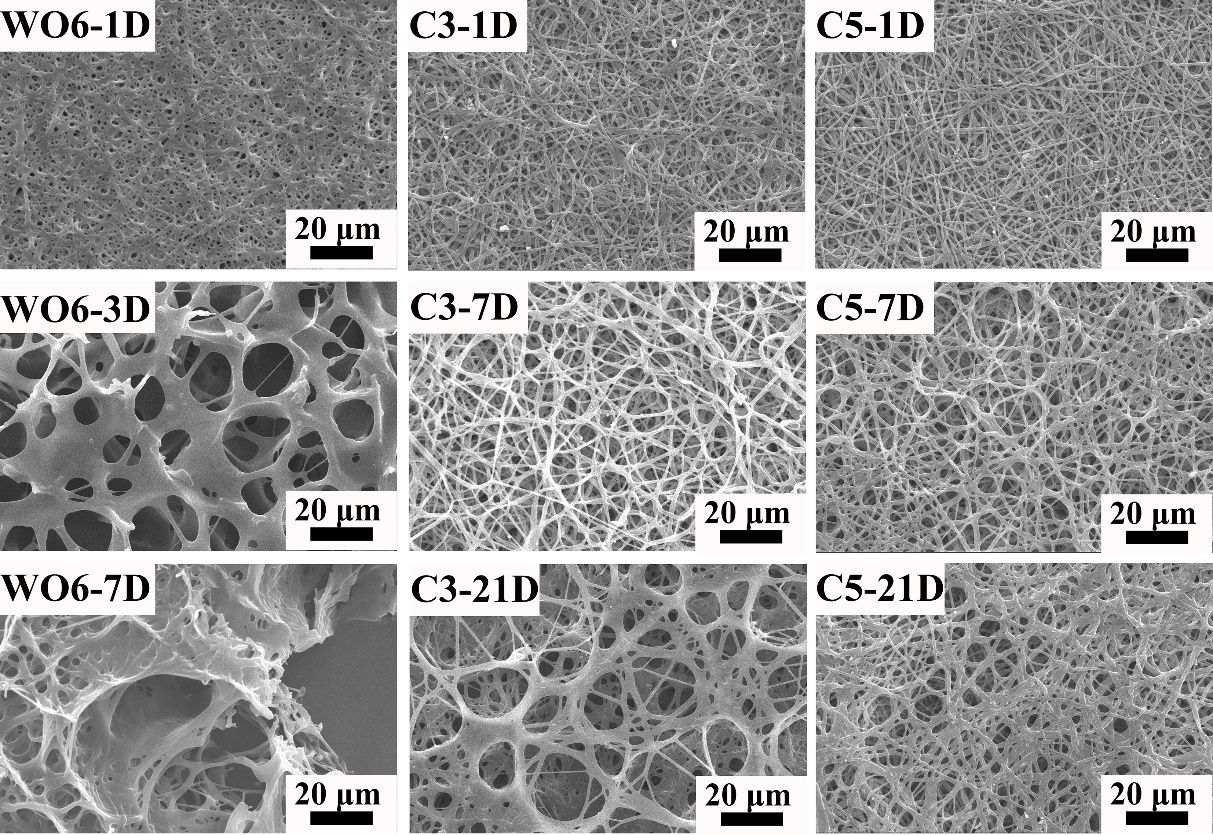


Figure S3 SEM images of WO6, C3, and C5 fibrous membranes after immersion in PBS for a certain time.

Figure S3 shows the morphology of crosslinked and uncross-linked WPU fibers after being degraded. After three days, the WO6 fibers fused with each other, and began to rupture. At the same time, the aperture significantly increased. The fibers severely broken into fragments and fused with each other after seven days. However, the crosslinked C3 and C5 fibers remained intact fiber morphology after seven days. After three weeks, the porosity of the crosslinked fibrous membrane also increased significantly.


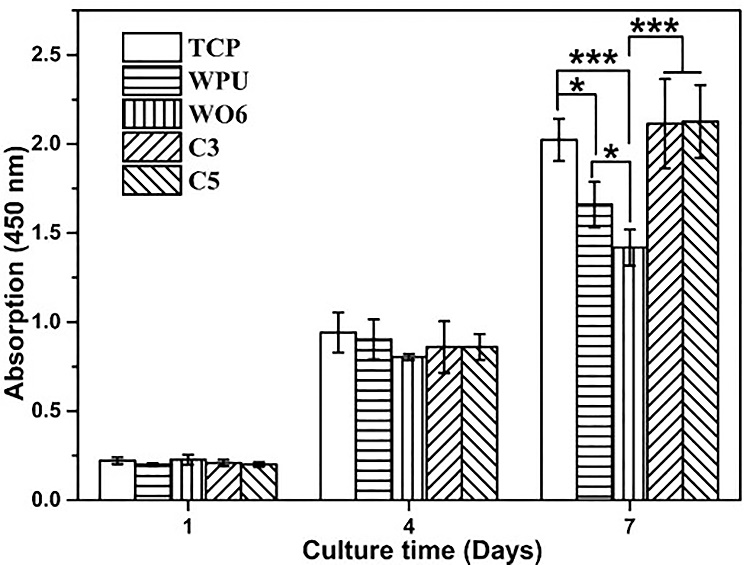


Figure S4 Cell proliferation study of rMSCs cultured on WO, C3, C5 fibrous films and WPU casting film, TCP as blank control (n = 5, ***p < 0.001, **p < 0.01, *p < 0.05).


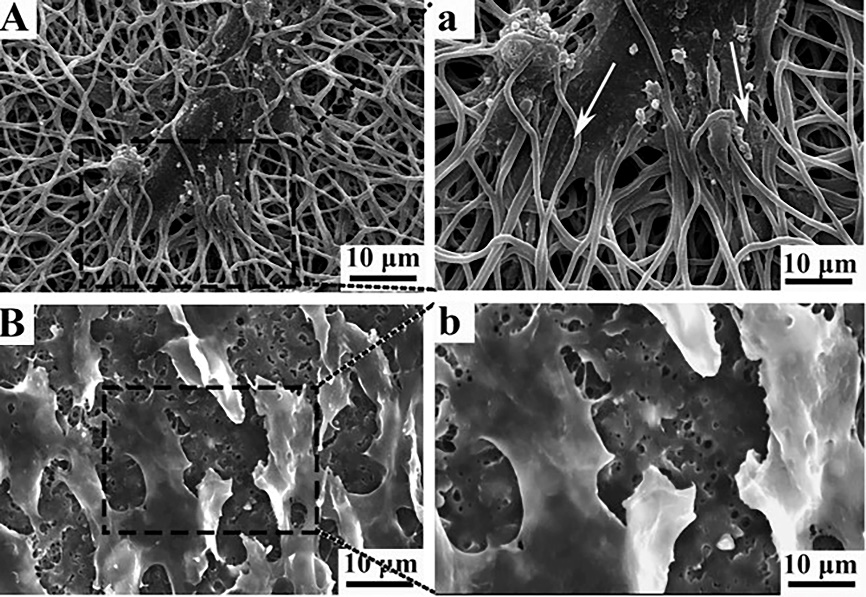


Figure S5 – SEM images of rMSCs on (A, a) C3 fibrous and (B, b) WPU casting film after cultured 4 days.

The viability and proliferation behavior of rMSCs cultured on the membrane was examined by CCK-8 kit. AS shown in Figure S4,no significant difference between each group at 1 and 4 days. At 7th day, the OD values of the cells on the cast film WPU and WO6 fibers were significantly lower than that of the blank control group ( TCP, *p* < 0.05) and the crosslinked fibrous membranes C3 and C5 ( *p* < 0.001). The result indicate that stable C3 and C5 fibrous membranes were more conducive to cell adhesion and proliferation than uncross-linked fiber membranes and casting film.

As shown in Figure S5A & a, the rMSCs inoculated on C3 fibers were polygonal and elongated. A large number of filopodia and plate podia stretched out at the edges. The cells were plump and expanded cytoplasm, shown by arrows in Figure S5a. However, the cells cultured on the casting film only had few pseudopods, which could not adhere to the substrate firmly, as shown in Figure S5B & b.
